# Cascading Effects of Root Microbial Symbiosis on the Development and Metabolome of the Insect Herbivore *Manduca sexta* L.

**DOI:** 10.3390/metabo11110731

**Published:** 2021-10-25

**Authors:** Dimitra Papantoniou, Fredd Vergara, Alexander Weinhold, Teresa Quijano, Bekzod Khakimov, David I. Pattison, Søren Bak, Nicole M. van Dam, Ainhoa Martínez-Medina

**Affiliations:** 1 German Centre for Integrative Biodiversity Research (iDiv) Halle-Jena-Leipzig, Puschstrasse 4, 04103 Leipzig, Germany; dimitra.papantoniou@idiv.de (D.P.); fredd.vergara@idiv.de (F.V.); alexander.weinhold@idiv.de (A.W.); 2 Institute of Biodiversity, Friedrich-Schiller Universität Jena, Dornburger Str. 159, 07743 Jena, Germany; 3 Departamento de Ecología Tropical, Campus de Ciencias Biológicas y Agropecuarias, Universidad Autónoma de Yucatán, Apartado Postal 4-116, Itzimná 97000, Mexico; esateresa@gmail.com; 4 Department of Food Science, University of Copenhagen Rolighedsvej 26, 1958 Frederiksberg C, Denmark; bzo@food.ku.dk; 5 Department of Plant and Environmental Sciences, Faculty of Science, University of Copenhagen, Thorvaldsensvej 40, 1871 Frederiksberg C, Denmark; dip@plen.ku.dk (D.I.P.); bak@plen.ku.dk (S.B.); 6 Plant-Microorganism Interaction, Institute of Natural Resources and Agrobiology of Salamanca, 37008 Salamanca, Spain

**Keywords:** arbuscular mycorrhizal fungi, *Trichoderma*, *Manduca sexta*, *Solanum lycopersicum*, LC–qToF–MS, metamorphosis

## Abstract

Root mutualistic microbes can modulate the production of plant secondary metabolites affecting plant–herbivore interactions. Still, the main mechanisms underlying the impact of root mutualists on herbivore performance remain ambiguous. In particular, little is known about how changes in the plant metabolome induced by root mutualists affect the insect metabolome and post-larval development. By using bioassays with tomato plants (*Solanum lycopersicum*), we analyzed the impact of the arbuscular mycorrhizal fungus *Rhizophagus irregularis* and the growth-promoting fungus *Trichoderma harzianum* on the plant interaction with the specialist insect herbivore *Manduca sexta*. We found that root colonization by the mutualistic microbes impaired insect development, including metamorphosis. By using untargeted metabolomics, we found that root colonization by the mutualistic microbes altered the secondary metabolism of tomato shoots, leading to enhanced levels of steroidal glycoalkaloids. Untargeted metabolomics further revealed that root colonization by the mutualists affected the metabolome of the herbivore, leading to an enhanced accumulation of steroidal glycoalkaloids and altered patterns of fatty acid amides and carnitine-derived metabolites. Our results indicate that the changes in the shoot metabolome triggered by root mutualistic microbes can cascade up altering the metabolome of the insects feeding on the colonized plants, thus affecting the insect development.

## 1. Introduction

Plants are a nutritious food source for insect species that belong to a wide range of taxonomic groups. To defend themselves against phytophagous insects, plants can produce a large range of metabolites that have repellent, anti-nutritive, or toxic effects on herbivores [1]. Commonly, they are secondary plant metabolites, such as alkaloids, benzoxazinoids, glucosinolates, terpenoids, phenolics, non-protein amino acids and cyanogenic glucosides [2,3]. Moreover, plants produce a number of defense proteins that reduce the digestibility of plants, such as protein inhibitors, α-amylase inhibitors, and polyphenol oxidases [4]. These metabolites can be produced constitutively or are induced upon herbivore damage. Many of these metabolites are either toxic or deterrent to herbivores, thereby reducing herbivore damage. In addition, some metabolites can also disrupt molting and other developmental and physiological processes in insects with lethal consequences [5,6,7]. For instance, alkaloids can affect nerve signal transmission in insects by disturbing the cell membrane and the cytoskeletal structure, causing the collapse and leakage of cells [8]. In addition, terpenoids can disturb the nervous system of insects by inhibiting acetylcholinesterase [9]. Furthermore, they can inhibit ATP synthase, the alkylation of nucleophiles and interfere with insect molting [10]. Many herbivores have evolved mechanisms to overcome the negative effects of plant defense metabolites. These include, among others, the rapid excretion and detoxification through conjugation and breakdown [11]. Moreover, specialist herbivores can also sequester some defensive chemicals and use them for self-defense against their own natural enemies [12,13,14]. As a result, specialized herbivores feeding on defended plants can ingest plant-produced toxic metabolites without suffering major consequences. Remarkably, anti-herbivore metabolites that are affecting basic processes, such as molting, are less easy for insect herbivores to adapt to, and therefore can also affect specialists.

In addition to detrimental interactions, mutualistic plant interactions are also frequent in nature [15]. Plants nurture a vast community of mutualistic microbes in their root systems, which provides their host plants with essential functions related to nutrient acquisition and immune system modulation. Among the most abundant and widespread root associated mutualistic microbes are the arbuscular mycorrhizal (AM) and *Trichoderma* fungi, which establish root symbioses with the majority of land plants [16,17]. Mycorrhizal fungi have co-evolved with plants for 400 million years and form symbiotic interactions with ≈80% of all plant species [18,19,20,21]. Mycorrhizal roots form an extensive network of fungal hyphae, which increases the plant’s exploratory capacity for water and mineral nutrients. Specialized fungal structures, called arbuscules, develop within root cells to facilitate nutrient exchange between the partners [20,21]. *Trichoderma* fungi are also ubiquitous soil microbes that display relatively low host specificity. In contrast to AM fungi, *Trichoderma* fungi are highly opportunistic, being capable of colonizing not only plant roots, but also aboveground plant parts and numerous other substrates such as wood and even other fungi [17,22,23,24,25].

Plants colonized by AM or *Trichoderma* fungi usually benefit from improved water and nutrient uptake, showing increased plant size, vigor, and nutrient levels [19,26,27,28,29]. These traits are important indicators of plant quality for herbivores [30,31,32]. In addition, root colonization by AM and *Trichoderma* fungi triggers a variety of molecular and biochemical responses in their host plants, including differential expression of defense-related genes and changes in the production of secondary metabolites. For instance, mycorrhizal colonization leads to a higher amount of iridoid glycosides and flavonoids in plantain and white clover leaves, respectively [33,34]. In addition, AM colonization has been shown to upregulate several plant secondary metabolite pathways, such as the carotenoid, the phenylpropanoid, and the antioxidant pathways [35,36], as well as to prime the accumulation of alkaloids and fatty acids upon herbivory [37]. Along the same lines, several studies have revealed that *Trichoderma* inoculation can trigger a higher accumulation of alkaloids, flavonoids, and phenolics; stress- and defense-related hormones; and transcripts related to different hormonal pathways in different plant species [38,39,40,41,42,43]. As a consequence, root colonization by AM or *Trichoderma* fungi can affect the performance of insect herbivores, as well as the amount of herbivore damage received by plants. However, the impact of the symbiotic fungi on herbivore performance can range from negative to positive, making the outcome of the symbiosis highly context-dependent [44]. 

Studies addressing the mechanistic basis of how plant mutualistic microbes affect plant–insect interactions focus specifically on physiological and metabolic changes in the plant. By contrast, how changes in the plant metabolome triggered by plant mutualistic microbes can affect the metabolome of insect herbivores, and thus affect their life cycle, remains obscure. Here, we hypothesize that the impact of root colonization by AM and *Trichoderma* fungi on the plant metabolome can cascade up, thereby affecting the metabolome of insect herbivores that feed on the leaves of root-colonized plants. We further hypothesize that these changes in the herbivore’s metabolome will affect its life cycle. By using tomato (*Solanum lycopersicum*) and the specialist chewing insect *Manduca sexta* as a model system, we found that root colonization by *Rhizophagus irregularis* or *Trichoderma harzianum* negatively affected the metamorphosis success of larvae that fed on the leaves of root-colonized plants. We further found that root colonization by the mutualistic fungi significantly affected the metabolome of the leaves as well as that of fat body and guts of larvae feeding on the colonized plants. Overall, our results indicate that the impact of root-mutualistic microbes can cascade up the trophic food chain, affecting the plant metabolome as well as the metabolism and the development of insect herbivores.

## 2. Results

### 2.1. Root Colonization by Rhizophagus irregularis or Trichoderma harzianum Impaired Pupation and Adult Emergence of Manduca sexta Larvae

**Figure 1:**
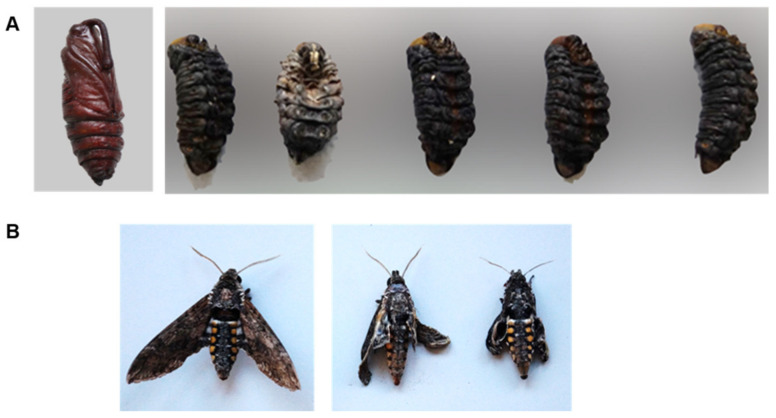
The impact of root colonization by *Rhizophagus irregularis* or *Trichoderma harzianum* on the morphology of the pupae and moths developed by *Manduca sexta* larvae that had fed from root-inoculated tomato plants. (**A**) Representative pupae with anomalous morphology developed by larvae that had fed from *T. harzianum*-inoculated plants (right panel), and a representative pupa presenting normal morphology developed by larvae that had fed form non-inoculated plants (left panel). (**B**) Representative *Manduca sexta* moths showing anomalous morphology (underdeveloped wings) formed by larvae that had fed from *R. irregularis*-inoculated plants (right panel) and a representative moth presenting normal morphology developed by larvae that had fed form non-inoculated plants (left panel).

We first aimed to assess whether root colonization by *R. irregularis* and *T. harzianum* affects the development of *M. sexta* larvae that fed on leaves of the root-inoculated plants. With this aim, we established a bioassay, in which we inoculated tomato roots with the mutualistic microbes and allowed *M. sexta* larvae to feed on the inoculated plants during the larval stage. After this, we assessed the impact of the different plant treatments on insect development, pupation, and adult emergence. We observed neither significant differences in the time required for the larvae to enter the pre-pupal stage, nor in the time required to complete the pupation process (Table 1). However, the number of individuals that successfully developed into normal pupae differed among the plant treatments. About 75% of the pupae developed by larvae fed on non-inoculated plants or *R. irregularis*-inoculated plants looked healthy (Table 1, Figure 1A). By contrast, about 60% of the larvae fed on *T. harzianum*-inoculated plants formed pupae with an aberrant morphology (Table 1, Figure 1A). Even though there were no significant differences in the pupal weight among the different plant treatments (Table 1), we observed significant differences in the sex ratio of the healthy pupae. Larvae fed on non-inoculated plants showed a female-based sex ratio of 0.69 (Table 1). The sex ratio of pupae emerging on *R. irregularis* plants was male-based (female: male ratio 0.3), being significantly different from that on control plants (χ^2^ = 4.196, α = 0.05). On *T. harzianum*-inoculated plants, the sex ratio was male-based as well, but the low number of healthy pupae recovered was not sufficient to detect statistically significant differences (Table 1). Most of the healthy pupae that were formed by individuals reared on control plants eclosed, and the emerged adults presented a normal morphology (Table 1, Figure 1B). By contrast, fewer healthy adults emerged from the individuals reared on *R. irregularis* plants and a higher number of adults showed an anomalous morphology, mostly underdeveloped wings (Table 1, Figure 1B). The number of healthy pupae formed by individuals reared on *T. harzianum*-colonized plants was not enough for detecting differences (Table 1, Figure 1B). In summary, the performance of individuals varied among the different plant treatments. About 53% of the surviving larvae that were reared on non-inoculated plants developed into apparently healthy adults. This percentage was much lower for individuals reared on *R. irregularis*-(27%) or on *T. harzianum* (15%)-inoculated plants. Our results thus indicate that root colonization by the mutualistic microbes has a strong effect on the metamorphosis success of *M. sexta* larvae that had fed on the leaves of the root-inoculated plants.

### 2.2. Root Colonization by Rhizophagus irregularis or Trichoderma harzianum Altered the Metabolomic Profile of Tomato Leaves

**Figure 2:**
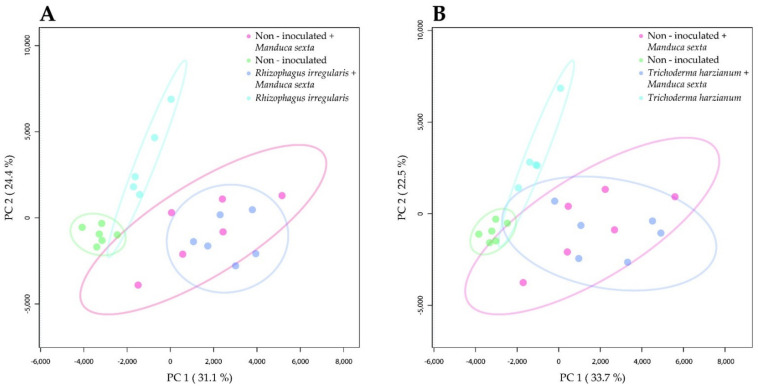
The impact of root colonization by *Rhizophagus irregularis* or *Trichoderma harzianum* and *Manduca sexta* shoot herbivory on the metabolome of tomato leaves. (**A**) Two-dimensional principal component analysis (PCA) scores plot of leaf samples of tomato plants that were non-inoculated or root-inoculated with *R. irregularis* and not challenged or shoot-challenged with *M. sexta* for three weeks. (**B**) PCA scores plot of leaf samples of tomato plants that were non-inoculated or root-inoculated with *T. harzianum* and not challenged or shoot-challenged with *M. sexta* for three weeks.

We next reasoned that the significant impact of the beneficial microbes on *M. sexta* pupation and adult emergence (Table 1) should be associated with changes in the leaf metabolome induced by the mutualistic microbes. To assess this, we performed an experiment, in which tomato plants were root-inoculated with *R. irregularis* or *T. harzianum*, and leaf challenged with *M. sexta* larvae. Three weeks after challenging the plants, we explored the impact of the mutualistic microbes and the herbivore on the constitutive and herbivore-induced leaf metabolome using liquid chromatography–quadrupole time of flight- mass spectrometry (LC-qToF-MS). The comparison between the *R. irregularis*-inoculated and non-inoculated plants showed that after three weeks of herbivory, the metabolomes of *R. irregularis*-inoculated and non-inoculated plants greatly overlapped (Figure 2A, dark blue dots vs. pink dots). However, in the absence of herbivory, the leaf metabolome of the *R. irregularis*-inoculated and non-inoculated plants clearly separated (Figure 2A, light blue dots vs. light green dots). Interestingly, the features that significantly contributed to the separation comprised alkaloids (identification level of confidence 1 according to the Metabolomics Standard Initiative (MSI) [45]). More precisely, the feature *m*/*z* = 578.4056, rt = 6.2 min was annotated as α-tomatine and showed its highest qToF intensity in the leaves of *R. irregularis*-inoculated plants (Figure S1A, Table S1). Similarly, the features *m*/*z* = 578.4051, rt = 6.0 min and *m*/*z* = 416.3524, rt = 6.0 min were annotated as α-tomatine isomers (level of confidence 1 according to MSI) and displayed their highest qToF intensity in the leaves of *R. irregularis*-inoculated plants (Figure S1B,C, Table S1). The feature *m*/*z* = 576.3898, rt = 5.5 min was annotated as an α-dehydrotomatine isomer (MSI level of confidence 2) and showed its highest qToF intensity in the leaf samples of the non-inoculated plants after herbivory (Figure S1D, Table S1). Moreover, the features *m*/*z* = 576.3896, rt = 5.6 min and *m*/*z* = 414.3369, rt = 5.6 min were annotated as α-dehydrotomatine isomers (MSI level of confidence 2) and displayed their highest qToF intensity in the leaves of *R. irregularis*-inoculated and non-inoculated plants under herbivory, but also in the leaves of *R. irregularis*-inoculated plants in the absence of herbivory (Figure S1E,F, Table S1).

The comparison between the *T. harzianum*-inoculated and non-inoculated plants showed that three weeks after herbivory, the metabolomes of *T. harzianum*-inoculated and non-inoculated plants greatly overlapped (Figure 2B, dark blue dots vs. pink dots). In absence of herbivory, a clear separation was observed between the *T. harzianum*-inoculated and the non-inoculated plants (Figure 2B, light blue dots vs. light green dots). The separation of the different treatments was driven by features such as steroidal glycoalkaloids. The feature *m*/*z* = 578.4056, rt = 6.2 min (MSI level of confidence 1) was predicted as α-tomatine and showed its highest qToF intensity in the leaves of *T. harzianum*-inoculated plants (Figure S2A, Table S2). Likewise, the features *m*/*z* = 578.4051, rt = 6.0 min and *m*/*z* = 416.3524, rt = 6.0 min were annotated as α-tomatine isomers (MSI level of confidence 1) and displayed their highest qToF intensities in the leaves of the plants inoculated with *T. harzianum* (Figure S2B,C, Table S2). In addition, the feature *m*/*z* = 414.3369, rt = 5.6 min was annotated as an α-dehydrotomatine isomer (MSI level of confidence 2) and showed the highest qToF intensity in the leaves of *T. harzianum*-inoculated plants (Figure S2D, Table S2). Another α-dehydrotomatine isomer, the feature *m*/*z* = 576.3896, rt = 5.6 min (MSI level of confidence 2) showed its highest qToF intensity in the leaves of non-inoculated and *T. harzianum*-inoculated plants after herbivory, but also in the leaves of *T. harzianum*-inoculated plants in the absence of herbivory (Figure S2E, Table S2). 

Overall, our results indicate that root inoculation with *R. irregularis* or *T. harzianum* alters the shoot metabolome of tomato plants, although this effect seems to be overruled by a long period of *M. sexta* herbivory. Our results further indicate that steroidal glycoalkaloids are prominently present among the leaf metabolites showing the highest variation due to the root-microbial inoculation, and that they accumulated in a greater proportion in the leaves of the root-inoculated plants.

### 2.3. Root Colonization by Rhizophagus irregularis or Trichoderma harzianum Altered the Metabolic Profile of the Manduca sexta Larval Gut

We next aimed to understand whether the impact of the mutualistic microbes on the leaf metabolome (Figure 2) cascades up, thereby affecting the herbivore’s metabolome. Therefore, we analyzed the metabolome of the caterpillars reared on the root-colonized plants by using ultra high performance liquid chromatography–mass spectrometry (UHPLC-MS). For conducting the analysis, we analyzed the metabolome of the gut and the fat body tissues separately. 

**Figure 3:**
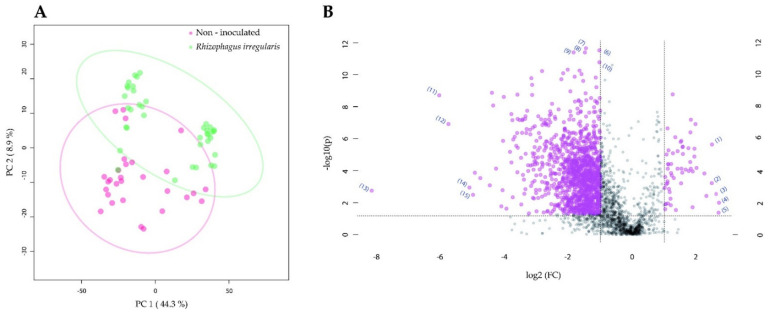
The impact of tomato root colonization by *Rhizophagus irregularis* on the gut metabolome of *Manduca sexta* larvae. (**A**) Two-dimensional principal component analysis (PCA) scores plot of *M. sexta* gut samples. (**B**) Significant metabolomic features annotated for the *M. sexta* gut samples. *Manduca sexta larvae* were fed for three weeks on leaves of tomato plants that were non-inoculated or root-inoculated with *R. irregularis*. In (**B**), the volcano plot displays fold change threshold (*x*-axis) of 2 and *t*-test threshold (*y*-axis) of 0.05. The purple circles represent features above the threshold. Both fold changes and *p*-values are log-transformed. The metabolomic features that showed the highest increase for the larvae reared on non-inoculated plants are found towards the right side of the volcano plot. The metabolomic features that showed the highest increase for the larvae reared on *R. irregularis*-inoculated plants are found towards the left side of the volcano plot. The most statistically significant metabolomic features for both treatments are found towards the top of the volcano plot.

When analyzing the gut tissue, we found that the metabolome of the larvae reared on non-inoculated plants did not separate from the metabolome of the larvae reared on *R. irregularis*-inoculated plants (Figure 3A). Still, we found several features, whose intensity significantly differed between the treatments. Among the annotated features that accumulated to a higher level in the gut tissue of larvae fed on non-inoculated plants, we found acyl-carnitines (*m*/*z* = 394.2951, rt = 12 min; *m*/*z* = 392.3157, rt = 17.9 min; and *m*/*z* = 378.3012, rt = 17.4) and glutathione (*m*/*z* = 308.0908, rt =1.1 min) (Figure 3B, Table 2; feature IDs 1, 3, 4, and 2, respectively; level of confidence of 3 for the acyl-carnitines and 2 for glutathione according to MSI). Among the annotated features that were indicated as significantly increased for the *R. irregularis*-inoculated plants, the amine piperidine (*m*/*z* = 86.0967, rt = 0.5 min, MSI level of confidence 2; Figure 3B, Table 2; feature ID 6) was found. In addition, the steroidal glycoalkaloid α-tomatine (*m*/*z* = 578.4056, rt = 8.4 min) was also annotated and was accumulated to higher levels in the gut tissue of larvae fed on *R. irregularis*-inoculated plants (*p* < 0.001) (Figure S3A; MSI level of confidence 1).

**Figure 4:**
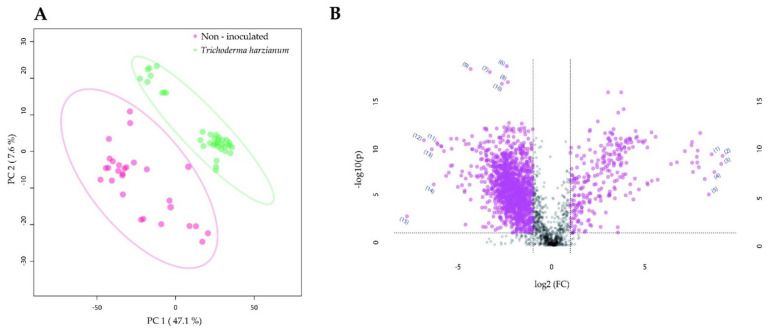
The impact of tomato root colonization by *Trichoderma harzianum* on the gut metabolome of *Manduca sexta* larvae. (**A**) Two-dimensional principal component analysis (PCA) scores plot of *M. sexta* gut samples. (**B**) Significant metabolomic features annotated for the *M. sexta* gut samples. *Manduca sexta* larvae were fed for three weeks on leaves of tomato plants that were non-inoculated or root inoculated with *T. harzianum*. In (**B**), the volcano plot displays fold change threshold (*x*-axis) of 2 and *t*-test threshold (*y*-axis) of 0.05. The purple circles represent features above the threshold. Both fold changes and *p*-values are log-transformed. The metabolomic features that showed the highest increase for the larvae reared on non-inoculated plants are found towards the right side of the volcano plot. The metabolomic features that showed the highest increase for the larvae reared on *T. harzianum*-inoculated plants are found towards the left side of the volcano plot. The most statistically significant metabolomic features for both treatments are found towards the top of the volcano plot.

The comparison of the gut metabolome between larvae fed on non-inoculated and *T. harzianum*-inoculated plants revealed a clear separation between the gut tissues (Figure 4A). The predicted features that were shown as significantly increased for the non-inoculated samples included compounds such as the acyl-carnitines, more precisely linoleyl-carnitine (*m*/*z* = 424.3421, rt = 13.9 min) and γ-linolenoyl-carnitine (*m*/*z* = 422.3274, rt = 13.2 min; MSI level of confidence 2) (Figure 4B, Table 3; feature IDs 2 and 5, respectively). Among the predicted features that were accumulated to a significantly higher level in the *T. harzianum*-inoculated samples, the acyl-carnitine *O*-palmitoyl-carnitine (*m*/*z* = 400.3421, rt = 14.8 min) was annotated (Figure 4B, Table 3; MSI level of confidence 2; feature ID 14). Moreover, α-tomatine (*m*/*z* = 578.4049, rt = 8.4 min) was also annotated and was indicated as significantly more highly accumulated (*p* <0.001) in the gut of *T. harzianum*-inoculated larvae (Figure S4A; MSI level of confidence 1).

### 2.4. Root Colonization by Rhizophagus irregularis or Trichoderma harzianum Altered the Metabolic Profile of the Manduca sexta Larval Fat Body 

**Figure 5:**
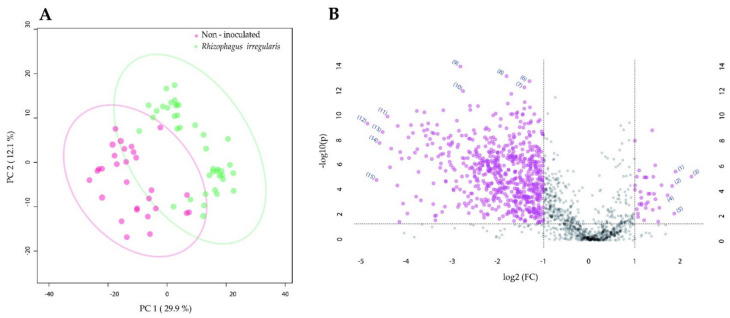
The impact of tomato root colonization by *Rhizophagus irregularis* on the fat body metabolome of *Manduca sexta* larvae. (**A**) Two-dimensional principal component analysis (PCA) scores plot of *M. sexta* fat body samples. (**B**) Significant metabolomic features annotated for the *M. sexta* fat body samples. *Manduca sexta* larvae were fed for three weeks on leaves of tomato plants that were non-inoculated, or root-inoculated with *R. irregularis*. In (**B**), the volcano plot displays fold change threshold (*x*-axis) of 2 and *t*-test threshold (*y*-axis) of 0.05. The purple circles represent features above the threshold. Both fold changes and *p*-values are log-transformed. The metabolomic features that showed the highest increase for the larvae reared on non-inoculated plants are found towards the right side of the volcano plot. The metabolomic features that showed the highest increase for the larvae reared on *R. irregularis*-inoculated plants are found towards the left side of the volcano plot. The most statistically significant metabolomic features for both treatments are found towards the top of the volcano plot.

We found that there was no clear separation between the fat body metabolome of *M. sexta* larvae reared on non-inoculated plants and that of larvae reared on *R. irregularis*-inoculated plants (Figure 5A). However, among the features that were found to accumulate to higher levels in the *R. irregularis*-inoculated samples, the compound (2*E*)-hexadecenoylcarnitine was annotated (*m*/*z* = 398.3265, rt = 13.7 min) (Figure 5B, Table 4; MSI level of confidence 2; feature ID 10). The steroidal glycoalkaloid alpha-tomatine (*m*/*z* = 578.4047, rt = 8.4 min) was also annotated, displaying higher levels of accumulation in the *R. irregularis*-inoculated larvae (*p* < 0.0001) (Figure S3B; MSI level of confidence 1).

**Figure 6:**
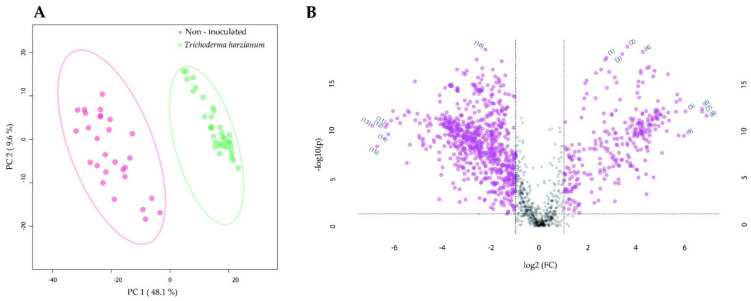
The impact of tomato root colonization by *Trichoderma harzianum* on the fat body metabolome of *Manduca sexta* larvae. (**A**) Two-dimensional principal component analysis (PCA) scores plot of *M. sexta* fat body samples. (**B**) Significant metabolomic features annotated for the *M. sexta* fat body samples. *Manduca sexta* larvae were fed for three weeks on leaves of tomato plants that were non-inoculated or root-inoculated with *T. harzianum*. In (**B**), the volcano plot displays fold change threshold (*x*-axis) of 2 and *t*-test threshold (*y*-axis) of 0.05. The purple circles represent features above the threshold. Both fold changes and *p*-values are log-transformed. The metabolomic features that showed the highest increase for the larvae reared on non-inoculated plants are found towards the right side of the volcano plot. The metabolomic features that showed the highest increase for the larvae reared on *T. harzianum*-inoculated plants are found towards the left side of the volcano plot. The most statistically significant metabolomic features for both treatments are found towards the top of the volcano plot.

The analysis of the fat body metabolome of the *M. sexta* larvae revealed a clear separation between the fat body metabolome of the larvae reared on non-inoculated plants and the metabolome of the larvae reared on *T. harzianum*-inoculated plants (Figure 6A). Among the features that were indicated as significantly higher accumulated for the non-inoculated samples, the compound octadecanamide (*m*/*z* = 284.2950, rt = 19.6 min; MSI level of confidence 2; feature ID 4; Figure 6B, Table 5) was annotated. In the *T. harzianum*-inoculated samples, the metabolomic features annotated as octanoic acid (*m*/*z* =145.1222, rt = 8.0 min) and benzamide (*m*/*z* = 625.2662, rt = 15.9 min) were accumulated to a higher level (Figure 6B, Table 5; MSI level of confidence 2; feature IDs 12 and 14, respectively). In addition, the steroidal glycoalkaloid α-tomatine (*m*/*z* = 578.4047, rt = 8.4 min) was annotated and accumulated in significantly higher levels in the fat body of *T. harzianum*-inoculated larvae (*p* < 0.0001; Figure S4B; MSI level of confidence 1).

## 3. Discussion

Root mutualistic microbes such as mycorrhiza and *Trichoderma* fungi can modulate the production of plant secondary metabolites and thus affect the performance of the herbivores feeding on the colonized plants [37,43]. Here, we demonstrate that the changes in plant metabolomes induced by root colonizing mutualistic microbes cascade up and modulate the metabolome of the insect herbivores feeding on leaves of root-colonized plants. Our results further indicate that these changes in the insect metabolome can affect the ability of the insect to successfully complete metamorphosis. Indeed, we first observed that root colonization by the mutualistic microbes *R. irregularis* or *T. harzianum* leads to the impairment of important stages of the insect metamorphosis process, particularly pupation and adult emergence. Specifically, we found that most of the larvae reared on *T. harzianum*-inoculated plants developed into abnormal pupae and displayed higher mortality rates. We further found that most of the larvae reared on *R. irregularis*-inoculated plants developed into moths with anomalous morphologies and did not emerge. Taking into consideration that healthy *M. sexta* adult females can lay over 200 eggs [46], the impact of the mutualistic microbes on the metamorphosis success is expected to strongly decrease population growth rates. Moreover, we found that herbivores feeding on *R. irregularis*-colonized plants showed a shift towards a male-biased sex ratio. The sex ratio of lepidopteran insects influences their reproductive potential [47]. In the case of *M. sexta*, it has been reported that the optimum sex ratio (female to male) that ensures high levels of oviposition, fecundity, and fertility lies between 0.5 and 0.67 [48]. We found that the sex ratio of insects feeding on non-inoculated plants was very similar to the optimal range (0.69), whereas the sex ratio of insects that fed on *R. irregularis*-colonized plants was much lower (0.3), thereby reducing the reproductive potential of the colony. These results may further indicate that female *M. sexta* individuals are more sensitive to the shoot alterations triggered by root colonizing microbes, compared to the male individuals. Accordingly, it has been reported that female herbivores are more sensitive to environmental disturbance and host quality compared to male individuals [48,49]. Overall, these results indicate that the impact of beneficial microbes on the performance of insect herbivores goes beyond the larval stage and can affect further stages of the herbivore life cycle, potentially having a strong impact on insect population dynamics.

It has been reported that the cumulative physiological effects experienced by insect herbivores during the larval feeding stages can impair the pupae formation, moths’ emergence, and even sex ratios [48,50,51]. In line with previous studies, our data indicated that root colonization by beneficial microbes altered the metabolome of the shoot of tomato plants [37,43,52]. Interestingly, we found several steroidal glycoalkaloids among the different features contributing strongly to the separation of leaf metabolomes of root-inoculated from non-inoculated plants. The abundance of steroidal glycoalkaloids was higher in leaves of plants inoculated with the mutualistic microbes. Steroidal glycoalkaloids in *Solanum* species function as prominent defense metabolites against herbivorous insects [53,54,55,56]. There are many reports demonstrating their toxicity and deterrence towards a wide range of herbivores [57,58,59,60,61]. Remarkably, besides affecting feeding and growth, steroidal alkaloids can affect other crucial physiological processes and interfere with insect development. It was suggested that steroidal glycoalkaloids might interact with the endocrine system of pests by interfering with hormone activity [62,63,64]. This is likely due to the fact that steroidal glycoalkaloids are derived from sterols and have similar chemical structures as the insect hormone ecdysone [65,66]. Hence, steroidal glycoalkaloids, through their similarity to sterols, are proposed to affect insect molting and metamorphosis, both processes that are regulated by ecdysone.

In addition to the strong effects of root colonization on herbivore development, we found an accumulation of the steroidal glycoalkaloid α-tomatine in the gut and fat body of herbivores reared on microbe-inoculated plants. It has been described that *M. sexta* has the ability to detoxify alkaloids, such as nicotine, most likely by degradation and excretion [67,68]. Typically, nicotine and many other alkaloids are absorbed by the midgut, pass into the haemolymph, are reabsorbed by the Malpighian tubules, and are finally excreted with the feces. Depending on the type of alkaloid, between 30 and 83% of them may be metabolized after absorption [68,69]. Although it could be that *M. sexta* is also able to degrade α-tomatine to some extent, our data indicated that the levels of α-tomatine in the gut and fat body of *M. sexta* were higher when the larvae were reared on plants that were colonized by the mutualistic microbes. Besides steroidal glycoalkaloids, we found a higher accumulation of piperidine in the gut of the larvae reared on *R. irregularis*-inoculated plants. Piperidine-containing alkaloids are complex structures that are derived from the amino acid *L*-lysine [70] and are able to disrupt the central nervous system of insects and mammals. For example, the piperidine alkaloids synthesized by the leguminous plant *Prosopis juliflora* have been reported to act as acetylcholinesterase inhibitors, butyrylcholinesterase inhibitors with Ca^2+^ channel blocking activity [71]. It is thus conceivable that enhanced levels of steroidal glycoalkaloids and piperidine alkaloids found in the insects that fed on the microbe-colonized plants might underlie, at least partially, the significant impact of the mutualistic microbes on *M. sexta* metamorphosis success.

Besides alkaloid-related metabolites, we found a higher accumulation of the metabolite benzamide in the fat bodies of larvae reared on *T. harzianum*-inoculated plants. Benzamides are amide derivatives of benzoic acid widely used as pesticides [72]. Treatment with benzamide chemicals has been related to an enhanced tissue lipid peroxidation and oxidative damage in juvenile catfish [73] and deformities in zebra fish embryos [74]. Our results thus suggest enhanced oxidative stress in the insects fed on *Trichoderma*-inoculated plants. Interestingly, we found a reduced accumulation of the metabolite glutathione in the gut of *M. sexta* larvae fed on microbe-inoculated plants. The primary role of glutathione (GSH) is to detoxify potentially deleterious substances and maintain the redox state in cells [75]. Insect glutathione S-transferases (GSTs) hold a pivotal role in detoxification and cellular antioxidant defense against oxidative stress by conjugating GSH to the electrophilic centers of natural and synthetic exogenous xenobiotics, including insecticides and allelochemicals [76,77]. Therefore, a reduced accumulation of glutathione in *M. sexta* larvae fed from microbe-inoculated plants might also reflect an enhanced oxidative stress, and thus a decreased fitness of the insects fed from the microbe-inoculated plants.

Our analyses further indicated that root colonization by the mutualistic microbes significantly altered the pattern of fatty acid amides in the gut and fat body of the insect. Although further analyses including standards would be required to unequivocally identify the altered metabolites, several studies indicate that fatty acid amides in the regurgitant of different species of lepidopteran larvae are important elicitors of plant defense responses [78,79,80,81]. The synthesis of fatty acid amides occurs by membrane-bound enzymes in the foregut and anterior midgut of the insect, utilizing insect-derived amino acids and host plant-derived fatty acids [78,82,83]. Even though it was demonstrated that they may play a complex role in nitrogen assimilation in insects [79], their exact physiological roles remain obscure [84]. Remarkably, we found that the root mutualists further triggered changes in insect fatty acids and lipids. For instance, we found a higher accumulation of octanoic acid in the fat body of larvae fed from *Trichoderma*-inoculated plants. Lipids are essential parts of cell and organelle membranes of insects; serve as important sources of energy; and act as precursors for secondary metabolites, waxes, pheromones, and defensive secretions [85,86,87]. Moreover, the metamorphosis process in *Sarcophaga argyrostoma* has been associated with specific changes in the profile of fatty acids, including octanoic acid [88]. Although further analysis would be required to establish solid conclusions, the changes in the fatty acid profile of the larvae triggered by the root-mutualistic fungi have the potential for altering the metamorphosis process of *M. sexta*.

In addition, we were able to predict the metabolite linoleyl-carnitine and other carnitine-derived metabolites in the gut and fat body of *M. sexta* larvae. It has been reported that carnitine-derived metabolites such as *L*-carnitine can affect the metabolism of lipids, proteins, and carbohydrates in mammals and zebrafish [89,90,91]. Moreover, carnitine and its acyl derivatives, which are involved in the lipid β-oxidation process, have been involved in the behavioral transition from the gregarious to solitary phase in locusts, possibly through modulating lipid metabolism and influencing the nervous system of the herbivore [92]. Although the ecological consequences of the altered levels of carnitine-derivatives in the body of *M. sexta* larvae may be different than in locusts, our results point to an important effect of root colonization by the mutualistic microbes on lipid metabolism.

In general, our study demonstrates that root colonization by the mutualistic microbes *R. irregularis* and *T. harzianum* impacts the metabolome of tomato shoots. These metabolomic alterations, triggered by the mutualistic microbes, affect the metabolome of *M. sexta* larvae feeding on the shoots of root-colonized plants. Among the most significant alterations, we found that *M. sexta* larvae feeding on the leaves of the root-colonized plants contain higher levels of the prominent steroidal glycoalkaloid α-tomatine. Moreover, feeding from microbial-colonized plants also led to changes related to oxidative damage, fatty acid metabolism, and lipid metabolism in the insect. Although further studies would be required to pinpoint the exact mechanism, we speculate that the microbe-triggered alterations in the insect metabolome underlie, at least partially, the impact of the root mutualists on insect metamorphosis.

## 4. Materials and Methods

### 4.1. Plant, Fungal, and Insect Material

Tomato (*Solanum lycopersicum*) cultivar Moneymaker was used in all the bioassays. Tomato seeds were obtained from Intratuin B.V (Woerden, the Netherlands). We used *Rhizophagus irregularis* as the AM fungus. The *R. irregularis* solid inoculum (INOQ-Sprint) was purchased from INOQ GmbH, Schnega, Germany (Available online: https://inoq.de/mykorrhiza-produkte/, accessed on 5 May 2021) with 220 mycorrhizal units per milliliter in sand. *Trichoderma harzianum* isolate T-78 (CECT 20714, Spanish Type Culture Collection) was maintained on potato dextrose agar (PDA, Sifin Diagnostics, Berlin, Germany) plates and regularly sub-cultured. The *T. harzianum* inoculum was prepared on a solid medium containing commercial oat and vermiculite [93]. Eggs from *M. sexta* (Lepidoptera, *Sphingidae*) were obtained from the Max Planck Institute for Chemical Ecology (Jena, Germany). The eggs and larvae were maintained in a plexiglass cage under 27 °C, 16 h light/8 h dark, and 50% relative humidity, and fed with artificial diet [94].

### 4.2. Plant Growth Conditions and Fungal Inoculation

Tomato seeds were surface-sterilized in 10% (*v*/*v*) sodium hypochlorite (NaOCl, 12% ChemSolute, Th. Geyer, Berlin, Germany) for 4 min, then thoroughly rinsed in water. After sterilization, the seeds were placed on fine-grained moist vermiculite and germinated in the dark for 3 days at 28 °C, followed by 7 days in a plant growth chamber at 25 ± 3 °C, 16 h/8 h day: night cycle, 65–70% RH conditions. Ten days after germination, the seedlings were transplanted into 400 mL pots containing a sterile sand/vermiculite mixture (1:1, *v*/*v*). Inoculation with *R. irregularis* was performed by mixing the *R. irregularis* inoculum with the sand/vermiculite mixture at 10% (*v*/*v*) before transplanting [95]. Inoculation with *T. harzianum* was achieved by mixing the *T. harzianum* inoculum with the substrate to achieve a final density of 1 × 10^6^ conidia g^−1^ before transplanting [95]. The plants were then placed in a completely randomized design in controlled climate chambers at 16 h/8 h day/night cycle, 65–70% RH. The plants were watered with tap water every second day, and once per week with Hoagland nutrient solution 50% water diluted [96]. Four weeks after transplanting, plants were used for the experiments. We did not find differences in the shoot biomass of the different treatments, and all the plants appeared similar, with no evident differences between the treatments.

### 4.3. Rhizophagus irregularis and Trichoderma harzianum Root Colonization

We confirmed that *R. irregularis* had efficiently colonized tomato roots in all the performed bioassays by incubating washed roots in 10% KOH (≥85% p.a., ROTH, Karlsruhe, Germany) and subsequently staining the fungal structures with 0.05% trypan blue (ROTH) [97]. The percentage of total root colonization was determined by the gridline intersection method [98] using a binocular stereo microscope (Leica DM 4000 B LED). The extent of mycorrhizal colonization was about 30–40% (percentage of total root length colonized by *R. irregularis*) in all the performed bioassays. We confirmed that *T. harzianum* had efficiently colonized the potting substrate in all the performed bioassays by using the plate count technique and PDA amended with 50 mg L^−1^ rose bengal (Applichem, Darmstadt, Germany) and 100 mg L^−1^ streptomycin sulphate (ROTH) [93]. Plates were incubated at 28 °C in darkness, and colony-forming unite (CFUs) were counted after 5 days. We found that the number of *T. harzianum* CFU in the potting media was similar to initial inoculation values in all the performed bioassays.

### 4.4. Bioassay for the Assessment of the Impact of Rhizophagus irregularis and Trichoderma harzianum on Manduca sexta Pupation and Adult Emergence

We placed a total of 25 *M. sexta* neonates on 5 plants (5 larvae per plant) of every treatment (inoculated with *R. irregularis* or *T. harzianum*, or non-inoculated). The larvae were placed on the third and fourth fully expanded leaf (counted from above) of every plant and allowed to feed freely on the entire plant. The plants were covered with fine mesh netting to prevent the escape of the larvae. After 7 days, the larvae were redistributed over different plants within the same treatment group (2 larvae per plant). During the bioassay, plants of the same treatment were exchanged for the damaged plants as needed to allow the larvae to feed ad libitum throughout the experiment. After 3 weeks, the surviving larvae were weighed and placed into plastic pupation boxes filled with vermiculite, which were placed in a growth chamber at 27 ± 3 °C, 16 h/8 h day/night cycle, and 50% RH. There, they were kept until they had reached the pre-pupal stage (approximately 3 days later). In the pupation boxes, the larvae were fed with artificial diet. The time required to reach the pre-pupal stage and the time required to complete the pupation process were recorded. After the pupation process had been completed, we visually checked the morphology of the developed pupae and recorded the number of pupae presenting morphological anomalies. From the pupae presenting a normal morphology, we collected data on pupal weight and sex determination. After the eclosion of the adults, we visually checked the morphology of the moths and recorded the number of moths presenting morphological anomalies. The number of pupae that failed to eclose was also recorded.

### 4.5. Bioassay for the Assessment of the Impact of Rhizophagus irregularis and Trichoderma harzianum on Tomato Leaf Metabolome

One first-instar *M. sexta* larva was placed on the apical leaflet of the third fully expanded leaf (counted from above) using a clip cage, which was moved to a new leaf every two days. The plants had been inoculated with *R. irregularis* or *T. harzianum*, or were non-inoculated, as described above. Dead *M. sexta* larvae were immediately replaced by larvae of the same age that had been feeding on plants from the same treatment. Empty clip cages were placed on leaves of non-infested plants. A total of six biological replicates (plants) were used per treatment and time point. Larvae were replaced weekly by new first-instar larvae to avoid consumption of the entire plant. Three weeks after first challenging the plants, the leaf the larvae were actually feeding on was collected, flash-frozen in liquid nitrogen, and stored at −80 °C for metabolomics analyses.

### 4.6. Bioassay to Assess the Impact of Rhizophagus irregularis and Trichoderma harzianum on the Metabolome of Manduca sexta Gut and Fat Body

We used 45 tomato plants per microbial treatment (inoculated with *R. irregularis* or *T. harzianum* or non-inoculated). On each plant, we placed 4 second-instar larvae on the third fully expanded leaf (counted from above) using a clip cage, which was moved to a new leaf every two days. Within the first week, dead larvae were removed and replaced by new ones of the same age. After the first week of herbivory, since the caterpillars had grown too large for the clip cage, we replaced the cage by a net bag wrapped around the plant. During the bioassay, plants of the same treatment were exchanged for the damaged plants as needed to allow the larvae to feed ad libitum throughout the experiment. Three weeks after the start of the experiment, the larvae were collected and kept on ice while transferred to be stored at −20 °C for dissection.

### 4.7. Leaf Metabolomics

#### 4.7.1. Extraction of Leaf Metabolites

We extracted leaf metabolites from 100 mg (fresh weight) of finely ground leaf material according to Rogachev and Aharoni [99,100], with the following modifications. For the sample extraction solution, high-performance liquid chromatography (HPLC grade, VWR International GmbH, Dresden, Germany) methanol and acetate buffer were used. For the preparation of the acetate buffer, 2.3 mL acetic acid (100% p.a., ROTH) and 3.41 g ammonium acetate (≥97% p.a., ACS, ROTH) were dissolved in 1L Millipore water. The pH of the buffer solution was adjusted to 4.8. For the extraction buffer, the acetate buffer (25%) was mixed with methanol (MeOH) (75%). For the extraction procedure, 100 ± 5 mg of each ground sample was weighed in a 2 mL Eppendorf tube. One milliliter of the extraction solution was added to each sample; the tube was tightly sealed and shaken for 10 s. Subsequently, each sample was sonicated for 5 min at 30 Hz using an FB 15061 sonicator (Elma, Singen, Germany) and then centrifuged for 15 min at 15,000× *g*. The supernatant was pipetted with a 1 mL pipette into a new-labeled Eppendorf tube, and the first Eppendorf tube was kept. By using the pipette, we added 1 mL of the extraction solution again to the pellet of the first extraction tube. The sealed tube was shaken again for 10 s and subsequently shaken for 5 min at 30 Hz in a sonicator. Then, it was centrifuged again for 15 min at 15,000× *g*. The two supernatants were combined, and the sample containing both supernatants was centrifuged for 10 min at 15,000× *g*; afterwards, 200 μL of the sample was transferred in an HPLC vial, and 800 μL of the extraction solution was added, resulting in a 1:5 dilution of the samples. The samples were then transferred to liquid chromatography–quadrupole time of flight–mass spectrometry for analysis.

#### 4.7.2. Liquid Chromatography–Quadrupole Time of Flight–Mass Spectrometry and Data Analysis of the Leaf Extracts

We obtained mass spectra of separated metabolites from the leaf samples by using UPLC–MS. The liquid chromatograph (Dionex UltiMate™ 3000, Thermo Scientific, Sunnyvale, CA, USA) was equipped with a C18 analytical column (Acclaim TM RSLC 120; 2.1 × 150 mm, 2.2 µm particle size, 120 Å pore size). The column was kept at 40 °C. The mobile phase was composed of solvent A, water/formic acid (99–100%, VWR) (0.05% *v*/*v*), solvent B, and acetonitrile (HPLC grade, VWR)/formic acid (0.05% *v*/*v*) at the flow rate of 400 μL min^−1^. The multi-step gradient for solvent B was 0–1 min, 5 %; 1–4 min, 28%; 4–10 min, 36%; 10–12 min, 95%; 12–14 min, 95%; 14–16 min, 5%; 16–18 min, 5%. The chromatograph was coupled to a maXis impact HD MS-qToF (Bruker Daltonics) operated in positive polarity. ESI source conditions were end plate offset = 500 V, capillary = 4500 V, nebulizer = 2.5 bar, dry gas = 11 L min^−1^, dry temperature = 220 °C. Transfer line conditions were funnels 1 and 2 = RF 300 Vpp, isCD energy = 0 eV, hexapole = 60 Vpp, quadrupole ion energy = 5 eV, low mass = *m*/*z* 50, collision cell energy = 10 eV, collision RF = 500 Vpp, transfer time = 60 µs, pre-pulse storage = 5 µs. The mass spectrometer operated with a mass range of *m*/*z* 50–1500 and a spectral acquisition rate of 3 Hz. Sodium formate clusters (10 mM) were used for calibrating the *m*/*z* values. The clusters mix consisted of 250 mL isopropanol (≥99% for LC–MS, VWR), 1 mL formic acid, and 5 mL 1M NaOH (≥99% p.a., ISO, ROTH), and the final volume was adjusted to 500 mL. For the identification of the steroidal glycoalkaloid α-tomatine in the leaf extracts, we used the commercial standard α-tomatine (500 mg, 0602, Lot: 12021719, Extrasynthase, Genay, France).

For the data analysis, Bruker .*d* files were transformed to .*abf* files using the program Abf Converter (https://www.reifycs.com/AbfConverter/, Tokyo, Japan, accessed on 5 May 2021). The .*abf* files were processed with the program MS-DIAL (v. 4.00, available online: http://prime.psc.riken.jp/compms/msdial/main.html, RIKEN, accessed on 5 May 2021) with the following parameters: data collection: mass accuracy: MS1 tolerance = 0.01 Da, retention time begin = 0.7 min, retention time end = 10 min, mass range begin = *m*/*z* 50, mass range end = *m*/*z* 1500; peak detection parameters: minimum peak height = 1000 amplitude, mass slice width = 0.1 Da, smoothing method = linear weighted moving average, smoothing level = 3 scans, minimum peak width = 5 scans; alignment parameter settings: retention time tolerance = 0.2 min, MS1 tolerance = 0.015 Da. We normalized the alignments against the total ion chromatogram. We exported the normalized data matrix containing all the alignments as a .*txt* file (spectra type = centroid). The level of confidence for the annotation/identification of the metabolomic features described in our analyses was determined on the basis of Malinowska and Viant [45].

### 4.8. Dissection of Manduca sexta Larvae and Separation of the Gut and Fat Body Samples

Before the dissection, the larvae were kept cool on ice for 30 min to sedate them. By using a sterile scalpel, each larva was dissected on two points. At first, the red horn at the bottom of the larval body was removed. Then, each larva was cut open at the ventral side from the head down to the posterior end using surgical scissors. For the separation of the samples, the central gut and the fat body tissues were carefully removed from the larval body using tweezers and subsequently separated one from each other. Guts and fat body samples from individual larvae were stored separately in a 1.5 mL safety lock Eppendorf tube, and the cap of each tube was punctured by using a sterile syringe before use. The samples were stored at −80 °C until further processing. Afterwards, the samples were freeze-dried and stored again at −80 °C until metabolite extraction.

### 4.9. Insect Metabolomics

#### 4.9.1. Extraction of Metabolites from the Gut and Fat Body of *Manduca sexta*

The gut and fat body sample dry masses were assessed before extraction. To handle the variability in the samples weight, we extracted the metabolites by following the ratio *w*/*v* = 1:2, meaning that in each sample tube, the volume of the extracting solution added in milliliters would be double that of the sample’s mass in milligrams. Using this procedure, we added the appropriate volume of 70% MeOH (≥99.9%, HiPerSolv CHROMANORM^®^ til HPLC, VWR, Søborg, Denmark), with 10 ppm palmitic acid methyl ester (Merck, Søborg, Denmark) and 10 ppm sorbitol (99%, Merck, Søborg) as internal standards, to each sample. The samples were vigorously vortexed for 30 s at 3000 rpm and then centrifuged at 12,000× *g* for 20 min at 4 °C. For LC–MS analyses, 50 μL of the supernatant (extracts) was transferred to a 200 μL glass insert in a 2 mL labelled glass vial. The vials (32 × 11.6 mm, Mikrolab Aarhus A/S, Denmark) containing the extracts were sealed with cap with septum and placed in the fridge (−20 °C) until analysis on LC–MS.

#### 4.9.2. Ultra High Performance Liquid Chromatography–Quadrupole Time of Flight–Mass Spectrometry and Data Analysis of the Insect Extracts

UHPLC–MS analyses were performed with the extracts of the gut and fat body of *M. sexta* larvae by using a Dionex Ultimate 3000RS UHPLC system (Thermo Fisher Scientific) system coupled to a Compact^TM^ (QqToF) mass spectrometer (Bruker Daltonics) with an electrospray ionization source. The UHPLC system was equipped with a DAD detector (acquiring from 190–800 nm), temperature-controlled auto-sampler (10 °C), and column oven (set to 40 °C).

Samples were analyzed on a Kinetex XB-C18 UHPLC column (100 × 2.1 mm, 1.7 μm, 100 Å pore size; Phenomenex) and eluted with a flow rate of 0.3 mL/min. The mobile phase composed of a gradient of solvent A (0.05 % formic acid (LC–MS, Ultra UHPLC–MS, Fluka, Rosklid, Denmark) in water) and solvent B (0.05% formic acid in acetonitrile (≥99.9%, HiPerSolv CHROMANORM^®^ til HPLC, suitable for UPLC/UHPLC, VWR, Søborg, Denmark)). The initial composition was 98% A and 2% B, which was held for 1 min before the composition of solvent B was increased linearly to 100% over 19 min. The column was washed with 100% solvent B for 7 min, before rapidly returning to 2% B over 1 min, and subsequently re-equilibrated at 2% B for 7 min prior to the next injection. The total run time was 35 min.

The qToF mass spectrometer was operated in full scan positive ion mode with the following instrument settings: *m*/*z* 50–1200: nebulizer gas (nitrogen), 2.0 bar; drying gas (nitrogen), 8 L/min; drying gas temperature, 220 °C; capillary voltage, 4000 V; full scan MS spectra acquisition rate, 2 Hz; set ion energy, 4.0 eV; set isolation mass, *m*/*z* 100.00; collision energy, 7.0 eV; and set collision cell RF, 500.0 Vpp. For the MS/MS spectra, the qToF mass spectrometer was operated as described previously with the following conditions for the collision cell (quadrupole): set ion energy, 4.0 eV; set isolation mass, *m*/*z* 100.00; collision energy, 7.0 eV; set collision cell RF, 500.0 Vpp. Every chromatogram was calibrated to provide accurate mass by automated infusion of sodium formate clusters (10 mM) at the beginning of each run. For the identification of the steroidal glycoalkaloid α-tomatine in the insect extracts, the commercial standard α-tomatine (CAS Number: 17406-45-0, Merck, Germany) was used. All data acquisition was automated using a combination of Chromeleon Express (Thermo Fisher Scientific), Compass qTOF Control (Version 4.0.15.3248, Bruker Daltonics), and Hystar (Version 3.2 SR4, Bruker Daltonics) software. For data analysis, DataAnalysis software (Version 4.3, Bruker Daltonics) was used. Metabolomic analyses were firstly performed using the Bruker MetaboScape Mass Spectrometry Software, version 5.0 (Build 683) (Bruker Daltonic GmbH, Bremen, Germany). The polarity mode was positive, and the workflow followed was T-ReX 3D (LC-QToF). The parameters set for the analyses were the following: minimum number of features for extraction = 4, presence of features in minimum number of analyses = 3, noise threshold = 2000, minimum peak length = 10, recursive feature extraction = 8, range for the retention time windows from 0.25 to 20 min, mass range from *m*/*z* 50 to 3000, ion deconvolution = [M+H]^+^, and EIC = 0.8. The chemical formula of the metabolites was automatically annotated by using the software’s Smart Formula option, and subsequently, each metabolite was annotated by using the spectral libraries and the MS/MS libraries available. The MS/MS libraries used have been created in international institutes on the basis of commercial standards.

### 4.10. Statistical Analysis

Pearson’s chi-squared test was used to determine whether there were statistically significant differences between the observed and expected frequencies of female and male *M. sexta* pupae that developed after feeding on the tomato plants of the three different treatment groups. One-way analysis of variance (ANOVA) was performed to evaluate the effect of the different treatments on the days required until pre-pupation, the days required for pupation, and the weight of the pupae showing normal morphology. For the statistical analysis of the tomato leaf data set, we used the web-based metabolomic data processing tool MetaboAnalyst (5.0) (available online: https://www.metaboanalyst.ca/, accessed on 5 May 2021) [101]. The data were filtered by taking the interquartile range and then pareto scaled. One-way analysis of variance (ANOVA) followed by Fisher’s least significant difference method (Fisher’s LSD) was performed in order to explain the variation within the data set. In addition, principal component analysis (PCA) was performed to identify significant differences among the treatments. For the statistical analyses of the *M. sexta* gut and fat body data set, firstly, the Bruker MetaboScape Mass Spectrometry Software (version 5.0) was used. A *t*-test was performed on the basis of the results of the processed spectral data. The resulting intensity table was exported and used for further statistical analysis using the web-based metabolomic data processing tool MetaboAnalyst (5.0) (available online: https://www.metaboanalyst.ca/, accessed on 5 May 2021) [101]. The data were filtered by interquartile range and transformed by using the generalized log transformation (glog 2). Pair-wise comparisons were then conducted between the non-inoculated and beneficial microbe-inoculated gut and fat body samples separately. These pair-wise comparisons included non-parametric *t*-tests with the level of significance set at α = 0.05 and volcano plots with a fold change threshold (*x*-axis) equal to 2 and *t*-test threshold (*y*-axis) equal to 0.05. The level of confidence for the annotation/identification of the metabolomic features described in all our analyses was determined on the basis of the work of Malinowska and Viant [45].

## Figures and Tables

**Table 1:** Impact of tomato root inoculation by *Rhizophagus irregularis* or *Trichoderma harzianum* on *Manduca sexta* pupation and adult emergence.

Treatment	No Larvae Reaching the Pre-Pupa Stage	Days Until Pre-Pupation (±SD)	Days until Pupation (±SD)	Total No of Pupae	No. of Pupae with Normal Morphology	No. of Pupae with Anomalous Morphology	Weight (g) of Normal Pupae (±SD)	F/M Ratio of the Normal Pupae	Number of Normal Pupae Not Eclosing	No of Moths with Anomalous Morphology	No of Moths with Normal Morphology
Non-inoculated	19	23.39 ± 1.47	22.06 ± 3.83	17	13	4	2.65 ± 0.49	9:4	2	1	10
*R. irregularis*	15	20.41 ± 2.54	19.94 ± 4.45	15	11	4	2.71 ± 0.61	3:8	3	4	4
*T. harzianum*	13	22.69 ± 1.9	11.61 ± 6.59	12	5	7	1.61 ± 0.91	2:3	1	2	2
One-way ANOVA	-	ns	ns	-	-	-	ns	χ^2^ = 4.196	-	-	-

One-way ANOVA evaluated the effect of the different treatments on the days required until pre-pupation, the days required for pupation, and the weight of the pupae showing a normal morphology. Pearson’s chi-squared test evaluated the effect of the different treatments on the ratio of normally developed female to male *M. sexta* pupae. SD: standard deviation, ns: not significant.

**Table 2:** The most significant metabolomic features annotated for the gut samples of *Manduca sexta* larvae fed for three weeks on leaves of tomato plants non-inoculated and root-inoculated with *Rhizophagus irregularis*. The feature number corresponds to the features given in Figure 3B, which are given by their corresponding mass to charge ratio (*m*/*z*) and retention time (rt) in minutes. The fold change (FC) refers to the feature’s qToF intensity and was calculated on the basis of a volcano plot conducted for the non-inoculated and *R. irregularis*-inoculated larvae. The volcano plot was conducted with a fold change threshold (*x*-axis) of 2 and *t*-test threshold (*y*-axis) of 0.05. The level of confidence of the identification/annotation is given according to Malinowska and Viant [45].

*Feature ID*	*Annotation*	*m*/*z*	*rt*	*FC*	*log_2_(FC)*	*Raw p-Value*	*Level of Confidence*
1	C_23_H_39_NO_4_	394.2951	12.0	5.63	2.5	*p* < 0.0001	3
2	C_10_H_17_N_3_O_6_S (Glutathione)	308.0908	1.1	5.61	2.49	*p* = 0.0006	2
3	C_24_H_41_NO_3_	392.3157	17.9	6.25	2.64	*p* = 0.002	3
4	C_23_H_39_NO_3_	378.3012	17.4	6.56	2.71	*p* = 0.01	3
5	C_27_H_39_O_3_	426.3009	17.9	6.49	2.7	*p* = 0.04	3
6	C_5_H_11_N (Piperidine)	86.0967	0.5	0.49	−1.03	*p* < 0.0001	2
7	C_55_H_94_N_10_O_16_P_2_	623.3105	8.0	0.37	−1.45	*p* < 0.0001	3
8	C_52_H_97_N_8_O_17_P_3_S	616.3028	8.0	0.36	−1.48	*p* < 0.0001	3
9	No formula	110.0653	1.2	0.28	−1.84	*p* < 0.0001	4
10	No formula	132.1018	0.4	0.49	−1.03	*p* < 0.0001	4
11	C_12_H_37_N_7_OS	328.2841	13.1	0.02	−6.05	*p* < 0.0001	3
12	C_23_H_43_N_3_O_6_P_2_	520.2708	14.9	0.02	−5.76	*p* < 0.0001	3
13	C_14_H_18_N_2_O_5_	295.1293	4.9	0.004	−8.16	*p* = 0.002	3
14	No formula	782.0187	8.2	0.03	−5.1	*p* = 0.001	4
15	No formula	644.2370	10.4	0.03	−4.5	*p* = 0.003	4

**Table 3:** The most significant metabolomic features annotated for the gut samples of Manduca sexta larvae fed for three weeks on leaves of tomato plants that were non-inoculated and root-inoculated with Trichoderma harzianum. The feature number corresponds to the features given in Figure 4B and are given by their corresponding mass to charge ratio (*m*/*z*) and retention time (rt) in minutes. The fold change (FC) refers to the feature’s qToF intensity and was calculated on the basis of a volcano plot conducted for the non-inoculated and T. harzianum-inoculated larvae. The volcano plot was conducted with a fold change threshold (*x*-axis) of 2 and *t*-test threshold (*y*-axis) of 0.05. The level of confidence of the identicication/annotation is given according to Malinowska and Viant [45].

Feature ID	Annotation	*m*/*z*	rt	FC	log_2_(FC)	Raw *p*-Value	Level of Confidence
1	C_23_H_39_NO_4_	394.2951	12.0	378.82	8.57	*p* < 0.0001	3
2	C_25_H_45_NO_4_ (linoleyl-carnitine)	424.3421	13.9	597.13	9.22	*p* < 0.0001	2
3	C_22_H_42_N_2_O	351.3370	12.9	614.71	9.26	*p*< 0.0001	3
4	No formula	224.2010	9.8	388.27	8.6	*p* < 0.0001	4
5	C_25_H_43_NO_4_ (γ-linolenoyl-carnitine)	422.3274	13.2	340.61	8.41	*p* < 0.0001	2
6	C_23_H_42_NO_7_P	476.2772	13.0	0.19	−2.4	*p* < 0.0001	3
7	C_13_H_16_N_4_	229.1444	7.9	0.1	−3.31	*p* < 0.0001	3
8	No formula	110.0653	1.2	0.2	−2.34	*p* < 0.0001	4
9	C_8_H_9_NO_2_	152.0709	6.7	0.05	−4.34	*p* < 0.0001	3
10	C_10_H_9_NO_4_	225.0872	1.3	0.16	−2.67	*p* < 0.0001	3
11	No formula	133.0866	7.9	0.01	−6.13	*p* < 0.0001	4
12	No formula	812.0668	8.0	0.009	−6.84	*p* < 0.0001	4
13	C_12_H_37_N_7_OS	328.2841	13.1	0.01	−6.42	*p* < 0.0001	3
14	C_23_H_45_NO_4_(*O*-palmitoyl-carnitine)	400.3421	14.8	0.01	−6.31	*p* < 0.0001	2
15	C_14_H_18_N_2_O_5_	295.1293	4.9	0.005	−7.74	0.0001	3

**Table 4:** The most significant metabolomic features annotated for the fat body samples of *Manduca sexta* larvae fed for three weeks on leaves of tomato plants that were non-inoculated and root-inoculated with *Rhizophagus irregularis*. The feature number corresponds to the features given in Figure 5B, which are given by their corresponding mass to charge ratio (*m*/*z*) and retention time (rt) in minutes. The fold change (FC) refers to the feature’s qToF intensity and was calculated on the basis of a volcano plot conducted for the non-inoculated and *R. irregularis*-inoculated larvae. The volcano plot was conducted with a fold change threshold (*x*-axis) of 2 and *t*-test threshold (*y*-axis) of 0.05. The level of confidence of the identification/annotation is given according to Malinowska and Viant [45].

*Feature ID*	*Annotation*	*m*/*z*	*rt*	*FC*	*log_2_(FC)*	*p-Value*	*Level of Confidence*
1	C_25_H_43_NO_5_	438.3210	11.9	3.71	1.89	*p* < 0.0001	3
2	C_32_H_66_N_4_O_7_P_2_S	357.2138	6.8	3.52	1.82	*p* < 0.0001	3
3	C_10_H_12_N_2_O_3_	209.0909	0.9	4.73	2.24	*p* < 0.0001	3
4	C_37_H_62_N_6_	591.5095	15.1	3.29	1.72	*p* < 0.0001	3
5	C_36_H_74_N_2_O_2_	567.5823	19.9	3.64	1.87	*p* < 0.008	3
6	C_23_H_4_NO_7_P	476.2774	11.7	0.4	−1.31	*p* < 0.0001	3
7	No formula	175.1163	1.1	0.37	−1.42	*p* < 0.0001	4
8	No formula	112.0502	1.0	0.28	−1.82	*p* < 0.0001	4
9	No formula	671.3542	5.9	0.14	−2.83	*p* < 0.0001	4
10	C_23_H_43_NO_4_ (2E)-hexadecenoylcarnitine)	398.3265	13.7	0.15	−2.77	*p* < 0.0001	2
11	No formula	224.2010	10.0	0.05	−4.42	*p* < 0.0001	4
12	C_37_H_66_N_8_O_10_	392.2516	6.9	0.03	−4.86	*p* < 0.0001	3
13	C_50_H_94_N_8_O_17_	360.5650	6.2	0.04	−4.53	*p* < 0.0001	3
14	C_41_H_79_N_14_O_5_PS	304.5352	5.9	0.04	−4.6	*p* < 0.0001	3
15	C_12_H_37_N_7_OS	328.2839	13.1	0.04	−4.66	*p* < 0.0001	3

**Table 5:** The most significant metabolomic features annotated for the fat body samples from *Manduca sexta* larvae fed for three weeks on leaves of tomato plants that were non-inoculated and root-inoculated with *Trichoderma harzianum*. The feature number corresponds to the features given in Figure 6B, which are given by their corresponding mass to charge ratio (*m*/*z*) and retention time (rt) in minutes. The fold change (FC) refers to the feature’s qToF intensity and was calculated on the basis of a volcano plot conducted for the non-inoculated and *T. harzianum*-inoculated larvae. The volcano plot was conducted with a fold change threshold (*x*-axis) of 2 and *t*-test threshold (*y*-axis) of 0.05. The level of confidence of the identification/annotation is given according to Malinowska and Viant [45].

*Feature ID*	*Annotation*	*m*/*z*	*rt*	*FC*	*log2(FC)*	*p-Value*	*Level of Confidence*
1	C_9_H_18_O_2_	159.1377	12.9	6.8	2.77	*p* < 0.0001	3
2	No formula	320.2558	14.5	12.36	3.63	*p* < 0.0001	4
3	C_17_H_31_NO	266.2483	13.6	10.71	3.42	*p* < 0.0001	3
4	C_18_H_37_NO (octadecanamide)	284.295	19.6	19.24	4.27	*p* < 0.0001	2
5	C_36_H_70_N_2_O_4_	595.5408	15.8	65.57	6.04	*p* < 0.0001	3
6	C_27_H_50_N_2_O_2_	435.3944	17.8	105.25	6.72	*p* < 0.0001	3
7	C_36_H_70_N_2_O_6_	627.5310	14.9	105.4	6.72	*p* < 0.0001	3
8	C_28_H_50_N_4_O_8_S	603.3416	10.4	119.75	6.9	*p* < 0.0001	3
9	C_15_H_41_N_6_O_3_PS	417.2771	10.6	62.57	5.97	*p* < 0.0001	3
10	C_23_H_42_NO_7_P	476.2774	11.7	0.21	−2.24	*p* < 0.0001	3
11	C_44_H_74_N_5_O_10_P	432.7648	8.0	0.01	−6.32	*p* < 0.0001	3
12	C_8_H_16_O_2_ (octanoic acid)	145.1222	8.0	0.01	−6.37	*p* < 0.0001	2
13	C_58_H_77_N_31_O_7_	660.8389	8.0	0.008	−6.93	*p* < 0.0001	3
14	C_35_H_36_N_4_O_7_ (benzamide)	625.2662	15.9	0.01	−6.26	*p* < 0.0001	2
15	No formula	742.0044	7.87	0.009	−6.73	*p* < 0.0001	4

## Data Availability

The authors are willing to share the data files of the study upon request. The data set was deposited in the open repository MetaboLights under the project number MTBLS2700 (available online: https://www.ebi.ac.uk/metabolights/).

## Supplementary Materials

The following are available online at https://www.mdpi.com/article/10.3390/metabo11110731/s1, Figure S1: qToF intensity of the metabolomic feature annotated as (**A**) α-tomatine; (**B**) an isomer of α-tomatine; (**C**) an isomer of α-tomatine; (**D**) α-dehydrotomatine; (**E**); an isomer of α-dehydrotomatine; (**F**) an isomer of α-dehydrotomatine. Samples correspond to leaves of tomato plants that were non-inoculated or root-inoculated with Rhizophagus irregularis and not challenged or shoot-challenged with Manduca sexta herbivory for three weeks. The qToF intensity was pareto scaled. The box plots summarize the scaled values of the qToF intensity in each group, the whiskers represent the values outside the middle 50%, the bisecting lines represent the median of the values, the yellow diamond indicates the mean qToF intensity in each group and the dots represent the samples in each group. Asterisks indicate statistically, Figure S2. qToF intensity of the metabolomic feature annotated as (**A**) α-tomatine; (**B**) an isomer of α-tomatine; (**C**) an isomer of α-tomatine; (**D**) an isomer of α-dehydrotomatine; (**E**) an isomer of α-dehydrotomatine. Samples correspond to leaves of tomato plants that were non-inoculated or root-inoculated with Trichoderma harzianum and not challenged or shoot-challenged with Manduca sexta herbivory for three weeks. The qToF intensity was pareto scaled. The box plots summarize the scaled values of the qToF intensity in each group, the whiskers represent the values outside the middle 50%, the bisecting lines represent the median of the values, the yellow diamond indicates the mean qToF intensity in each group and the dots represent the samples in each group. Asterisks indicate statistically significant differences among the groups according to Fisher’s Least Significance Difference (LSD) test (*p* ≤ 0.05)., Table S1. Results for the one-way ANOVA for the effect of root colonization by Rhizophagus irregularis on the qToF intensity of steroidal glycoalkaloids in the leaf metabolome of tomato plants after Manduca sexta herbivory for three weeks. Each metabolomic feature is given by its mass to charge ratio (*m*/*z*) and its retention time (rt). FDR: means False Discovery Rate., Figure S3. qToF intensity of the metabolomic feature annotated as α-tomatine in the gut (**A**) and fat body (**B**) samples of Manduca sexta larvae reared for three weeks on leaves of plants that were non-inoculated and root-inoculated by Rhizophagus irregularis. The qToF intensity was log transformed. The box plots summarize the values of the qToF intensity in each group, the whiskers represent the values outside the middle 50%, the bisecting lines represent the median of the values, the yellow diamond indicates the mean qToF intensity in each group and the dots represent the samples in each group. Asterisks indicate statistically significant difference between the treatments according to Student *t*-tests (*p* ≤ 0.05)., Figure S4. qToF intensity of the metabolomic feature annotated as α-tomatine in the gut (**A**) and fat body (**B**) samples of Manduca sexta larvae reared for three weeks on leaves of plants that were non-inoculated and root-inoculated by Trichoderma harzianum. The qToF intensity was log transformed. The box plots summarize the values of the qToF intensity in each group, the whiskers represent the values outside the middle 50%, the bisecting lines represent the median of the values, the yellow diamond indicates the mean qToF intensity in each group and the dots represent the samples in each group. Asterisks indicate statistically significant difference between the treatments according to Student *t*-tests (*p* ≤ 0.05). Table S2. Results for the one-way ANOVA for the effect of root colonization by Trichoderma harzianum on the qToF intensity of steroidal glycoalkaloids in the leaf metabolome of tomato plants after Manduca sexta herbivory for three weeks. Each metabolomic feature is given by its mass to charge ratio (*m*/*z*) and its retention time (rt). FDR: means False Discovery Rate.
