# Supplementary material for: Cascading Effects of Root Microbial Symbiosis on the Development and Metabolome of the Insect Herbivore Manduca sexta L
Source: Metabolites. 2021 Oct 25;11(11):731. doi: 10.3390/metabo11110731 (PMC8622251; doi:10.3390/metabo11110731)
Supplement: Supplementary file 1 [file metabolites-11-00731-s001.zip › metabolites-1309078-supplementary.pdf]

SUPPLEMENTARY MATERIAL

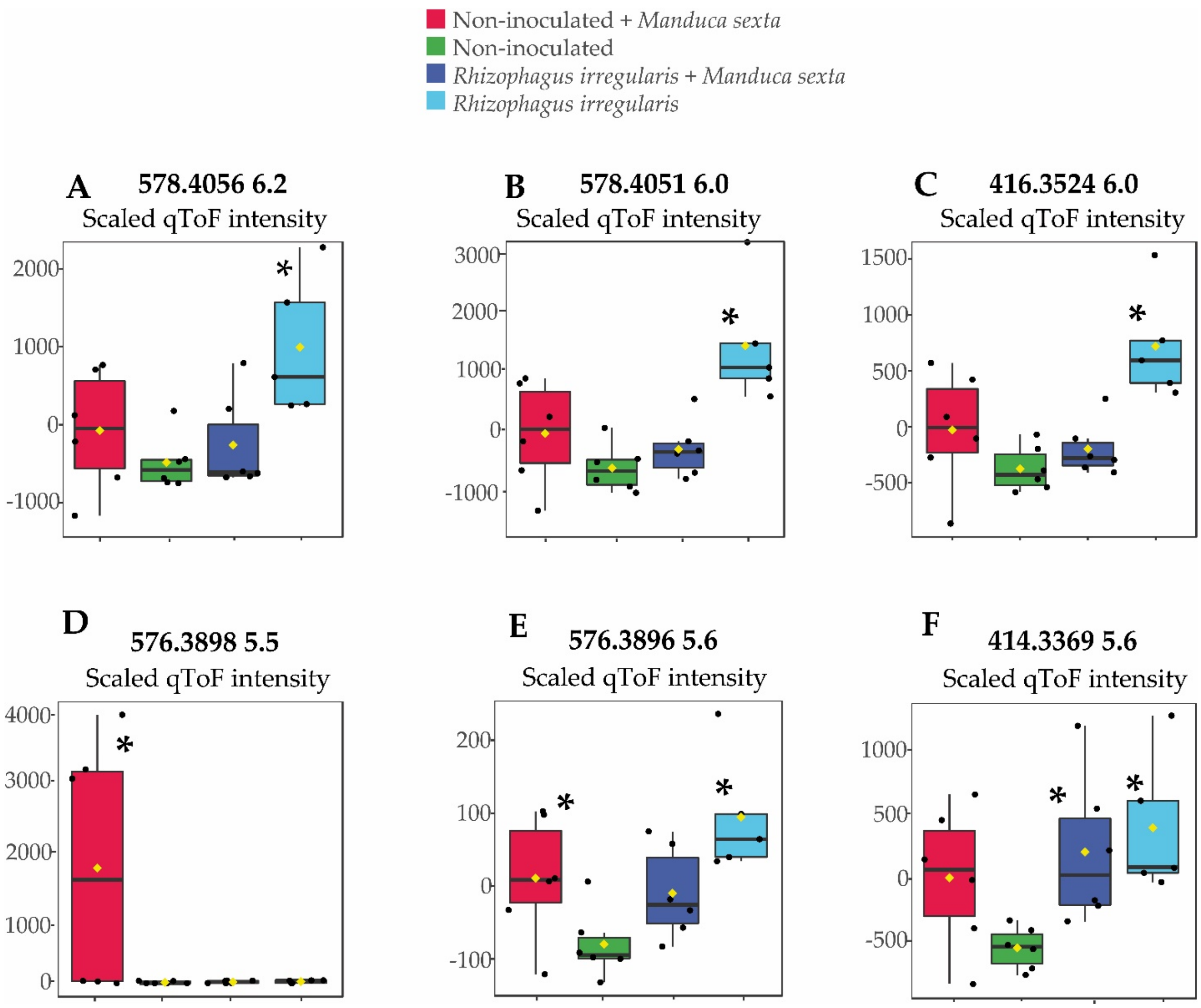

Figure S1. qToF intensity of the metabolomic feature annotated as (**A**) α-tomatine; (**B**) an isomer of α-tomatine; (**C**) an isomer of α-tomatine; (**D**) α-dehydrotomatine; (**E**); an isomer of α-dehydrotomatine; (**F**) an isomer of α-dehydrotomatine. Samples correspond to leaves of tomato plants that were non-inoculated or root-inoculated with *Rhizophagus irregularis* and not challenged or shoot-challenged with *Manduca sexta* herbivory for three weeks. The qToF intensity was pareto scaled. The box plots summarize the scaled values of the qToF intensity in each group, the whiskers represent the values outside the middle 50%, the bisecting lines represent the median of the values, the yellow diamond indicates the mean qToF intensity in each group and the dots represent the samples in each group. Asterisks indicate statistically

significant differences among the groups according to Fisher's Least Significance Difference (LSD) test ($p \leq 0.05$).

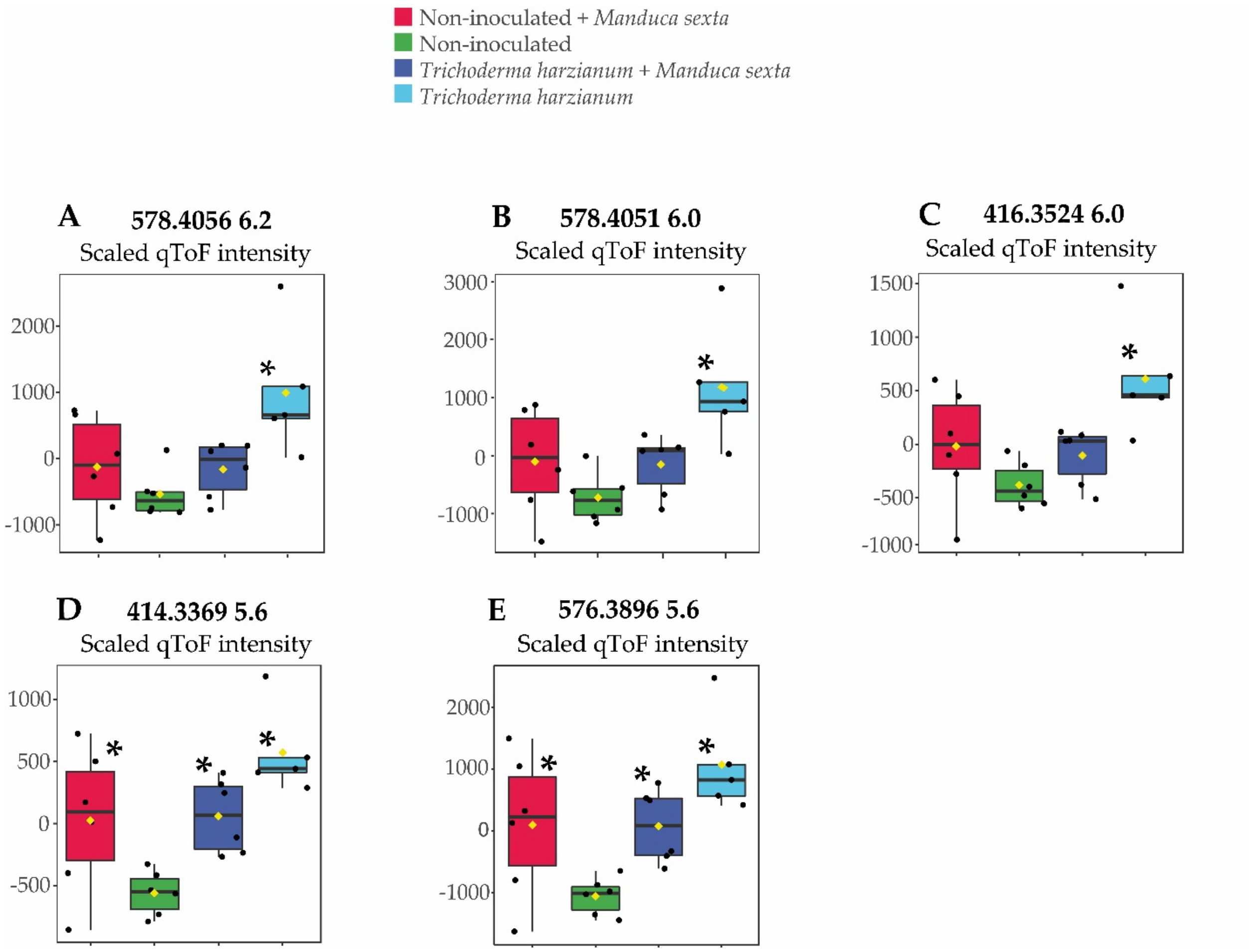

Figure S2. qToF intensity of the metabolomic feature annotated as (**A**) α-tomatine; (**B**) an isomer of α-tomatine; (**C**) an isomer of α-tomatine; (**D**) an isomer of α-dehydrotomatine; (**E**) an isomer of α-dehydrotomatine. Samples correspond to leaves of tomato plants that were non-inoculated or root-inoculated with *Trichoderma harzianum* and not challenged or shoot-challenged with *Manduca sexta* herbivory for three weeks. The qToF intensity was pareto scaled. The box plots summarize the scaled values of the qToF intensity in each group, the whiskers represent the values outside the middle 50%, the bisecting lines represent the median of the values, the yellow diamond indicates the mean qToF intensity in each group and the dots represent the samples in each group. Asterisks indicate statistically significant differences among the groups according to Fisher's Least Significance Difference (LSD) test ($p \leq 0.05$).

Table S1. Results for the one-way ANOVA for the effect of root colonization by *Rhizophagus irregularis* on the qToF intensity of steroidal glycoalkaloids in the leaf metabolome of tomato plants after *Manduca sexta* herbivory for three weeks. Each metabolomic feature is given by its mass to charge ratio (*m/z*) and its retention time (rt). FDR: means False Discovery Rate.

Feature (m/z, rt)	*F-value*	*p-value*	*FDR*
578.4056 6.2	4.97	0.01	0.02
578.4051 6.0	8.43	<0.001	0.003
416.3524 6.0	8.3	<0.001	0.003
576.3898 5.5	4.6	0.01	0.03

576.3896 5.6	5.7	0.006	0.01
414.3369 5.6	3.9	0.03	0.05

Table S2. Results for the one-way ANOVA for the effect of root colonization by *Trichoderma harzianum* on the qToF intensity of steroidal glycoalkaloids in the leaf metabolome of tomato plants after *Manduca sexta* herbivory for three weeks. Each metabolomic feature is given by its mass to charge ratio (*m/z*) and its retention time (rt). FDR: means False Discovery Rate.

Feature (m/z, rt)	*F-value*	*p-value*	*FDR*
578.4056 6.2	5.3	$p = 0.008$	0.02
578.4051 6.0	5.9	$p = 0.005$	0.01
416.3524 6.0	5.6	$p = 0.006$	0.02
414.3369 5.6	8.0	$p = 0.001$	0.004
576.3896 5.6	6.8	$p = 0.003$	0.008

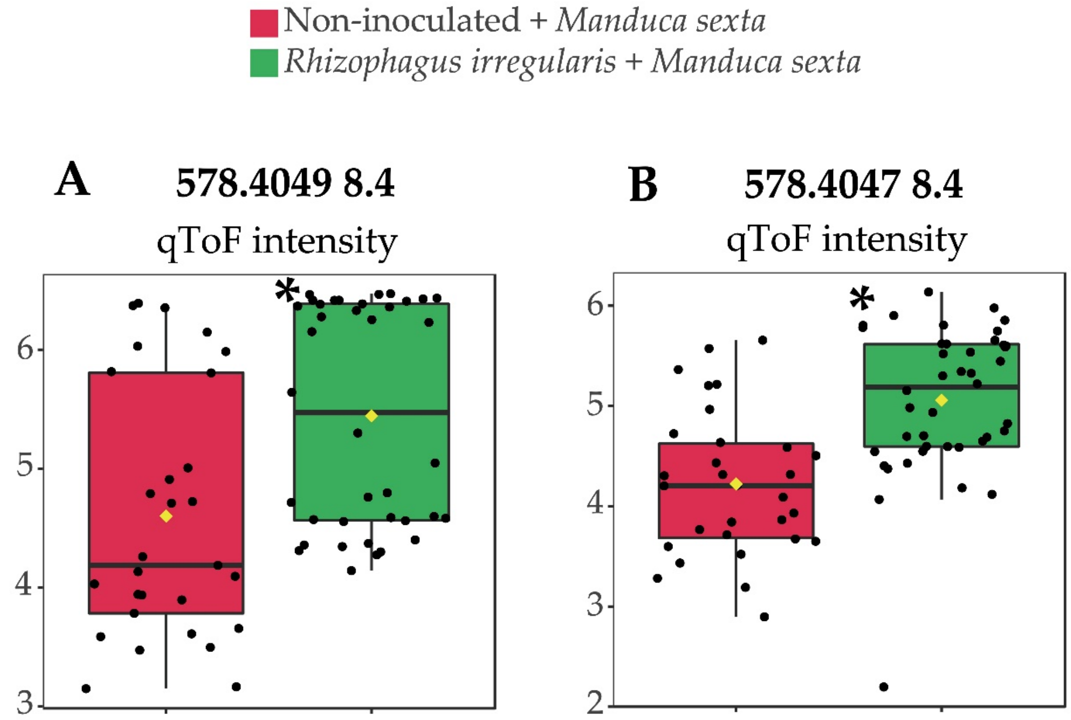

Figure S3. qToF intensity of the metabolomic feature annotated as α-tomatine in the gut (**A**) and fat body (**B**) samples of *Manduca sexta* larvae reared for three weeks on leaves of plants that were non-inoculated and root-inoculated by *Rhizophagus irregularis*. The qToF intensity was log transformed. The box plots summarize the values of the qToF intensity in each group, the whiskers represent the values outside the middle 50%, the bisecting lines represent the median of the values, the yellow diamond indicates the mean qToF intensity in each group and the dots represent the samples in each group. Asterisks indicate statistically significant difference between the treatments according to Student T-tests ($p \leq 0.05$).

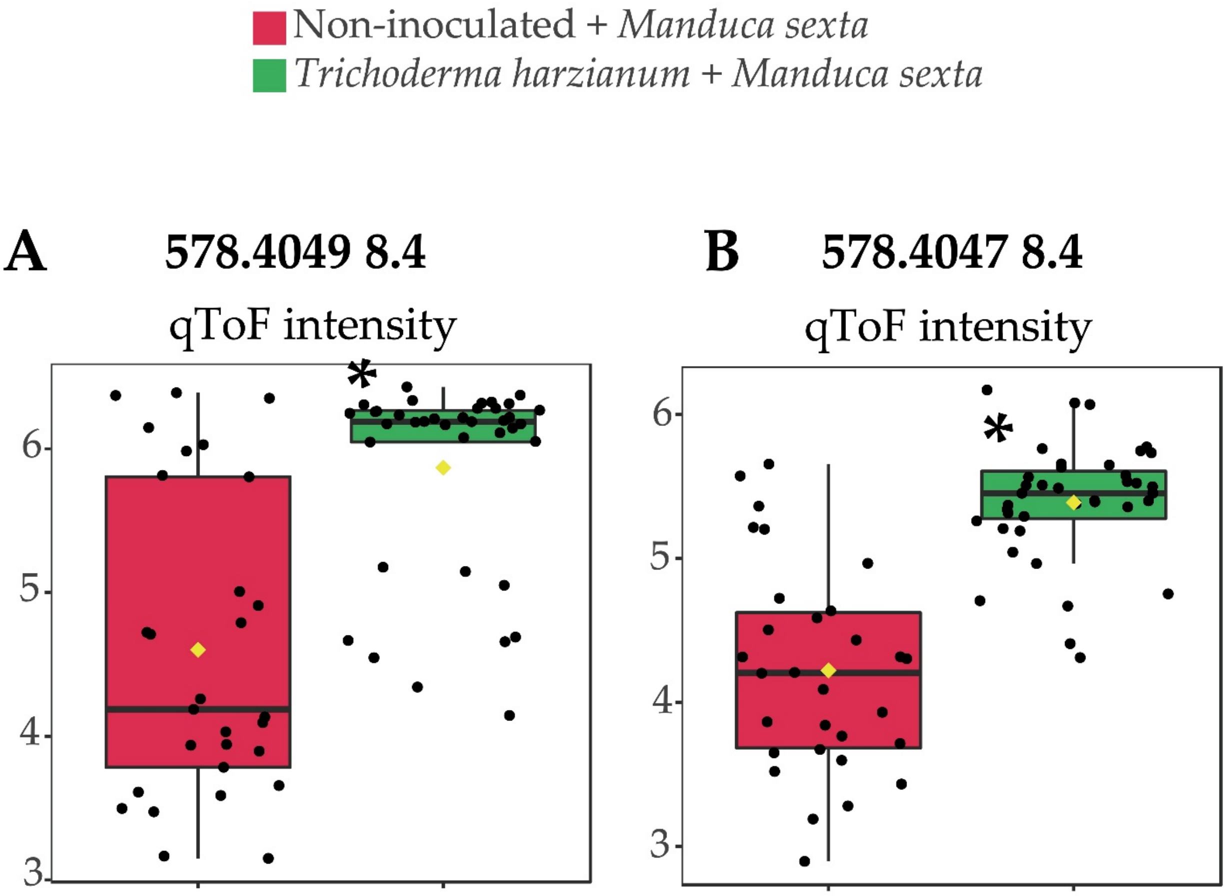

Figure S4. qToF intensity of the metabolomic feature annotated as α-tomatine in the gut (**A**) and fat body (**B**) samples of *Manduca sexta* larvae reared for three weeks on leaves of plants that were non-inoculated and root-inoculated by *Trichoderma harzianum*. The qToF intensity was log transformed. The box plots summarize the values of the qToF intensity in each group, the whiskers represent the values outside the middle 50%, the bisecting lines represent the median of the values, the yellow diamond indicates the mean qToF intensity in each group and the dots represent the samples in each group. Asterisks indicate statistically significant difference between the treatments according to Student T-tests ($p \leq 0.05$).
